# Powerline noise elimination in biomedical signals via blind source separation and wavelet analysis

**DOI:** 10.7717/peerj.1086

**Published:** 2015-07-02

**Authors:** Samuel Akwei-Sekyere

**Affiliations:** Neuroscience Program, Michigan State University, East Lansing, MI, USA; Department of Mathematics, Michigan State University, East Lansing, MI, USA

**Keywords:** Noise-assisted noise reduction, Electrophysiology, Neurotechnology, Ensemble empirical mode decomposition, Independent component analysis, Wavelet, Machine learning

## Abstract

The distortion of biomedical signals by powerline noise from recording biomedical devices has the potential to reduce the quality and convolute the interpretations of the data. Usually, powerline noise in biomedical recordings are extinguished via band-stop filters. However, due to the instability of biomedical signals, the distribution of signals filtered out may not be centered at 50/60 Hz. As a result, self-correction methods are needed to optimize the performance of these filters. Since powerline noise is additive in nature, it is intuitive to model powerline noise in a raw recording and subtract it from the raw data in order to obtain a relatively clean signal. This paper proposes a method that utilizes this approach by decomposing the recorded signal and extracting powerline noise via blind source separation and wavelet analysis. The performance of this algorithm was compared with that of a 4th order band-stop Butterworth filter, empirical mode decomposition, independent component analysis and, a combination of empirical mode decomposition with independent component analysis. The proposed method was able to expel sinusoidal signals within powerline noise frequency range with higher fidelity in comparison with the mentioned techniques, especially at low signal-to-noise ratio.

## Introduction

Powerline noise from recording biomedical devices have been known to introduce distortion to recorded signals and, as a result, compromise their integrity and negatively affect their interpretations. Consequently, fields such as neural engineering, neurosurgery, cardiology, and drug discovery need to refine raw data by eliminating powerline noise. An adulteration of neural signals by powerline noise makes it difficult to understand the properties of ion channels and how ensembles of neurons interact to perform specific computations for observed behaviors. In effect, clinical applications of neural interfaces such as brain-machine and brain-computer interfaces become difficult to implement. Neurosurgical procedures often involve recording neural activity for intraoperative monitoring, localization of brain regions, and proper placement of stimulating electrodes for deep brain stimulation (Finnis et al., 2003; Guridi et al., 1999; Schramm et al., 1990). Special rhythms of the brain such as high synchronous intra-cortical activity in the gamma frequency band (25 Hz to 100 Hz) often lead to dyskinesia (Brown, 2006). Since powerline noise exists within the frequency range of some of these important neural oscillations, it is essential to extinguish it to make valid neuro-pathological conclusions and, in succession, develop appropriate approaches for treatment. Details about the condition of the heart and, in turn, the flow of blood in the body is vital in disease diagnosis. By the same token, electrocardiography has become an indispensable clinical tool in cardiology. The electrocardiogram (ECG) is a representation of the electrical activity of cardiac tissue during systole and diastole. This signal is typically obtained by measuring the electrical potential between a specific spatial combination of recording electrodes. As the cardiac depolarization-mediated mean electrical vector approaches a positive electrode, the ECG signal increases positively in amplitude and vice versa. The P wave, QRS complex and T wave in ECG signals are manifestations of atrial depolarization, ventricular depolarization and ventricular repolarization respectively. Although its origin is still under debate, an inversion of the U wave has been shown to be an indicator of myocardial ischemia (Miwa et al., 1993; Gerson et al., 1979; Van Eck, Kors & van Herpen, 2005). In the presence of powerline noise, a malformation in the morphology of the ECG waveform—and by extension, the U wave—is very difficult to detect with the naked eye. Lastly, microelectrodes have been demonstrated to be very useful in drug discovery (Cui et al., 2006; Jones et al., 2011; Jain & Muthuswamy, 2008; Stett et al., 2003). Signal perturbations influenced by powerline noise make it difficult to understand drug-tissue interactions for the development of clinically-viable pharmaceutical products prior to clinical trials. For these reasons, the elimination of powerline noise has been a vigorous field of investigation (Keshtkaran & Yang, 2014; Agrawal & Gupta, 2013; Van Alste & Schilder, 1985; Levkov et al., 2005; Piskorowski, 2012; Poungponsri & Yu, 2013; Keshtkaran & Yang, 2012; Poornachandra & Kumaravel, 2008; Clancy, Morin & Merletti, 2002; Philips, 1996).

Powerline noise is characterized by a chronic sinusoidal 50/60 Hz element which can be observed in raw recordings of biomedical data. The sinusoidal component is usually a result of the use of devices that employ alternating current as a source of power. Alternating current has been used in the design of biomedical devices because it has been demonstrated to possess the quality of being relatively stable, especially over long distances, as opposed to direct current. In some cases, powerline noise is removed by low-pass filters with cut-off frequencies below 50/60 Hz. Although this approach solves the problem of extinguishing powerline noise and has its applications, it is challenging to implement on some forms of biomedical data because of the importance of broadband signals. These include, but are not limited to, electroencephalogram signals and extracellular neural recordings. For some purposes, such as the extraction of action potentials from broadband neural tissue recordings, the pitfall of obtaining noisy recordings can be potentially avoided by employing a high-pass filter with a cut-off frequency above 250 Hz (Oweiss, 2010). Band-stop filters have been used to attenuate powerline noise to inconspicuous levels. Nevertheless, due to the instability of biomedical signals, band-stop filters sometimes fail in reducing noise with 50/60 Hz center frequency and thus may have to rely on correction methods (Ferdjallah & Barr, 1994; Ferdjallah & Barr, 1990; Hamilton, 1996). Although band-stop Butterworth filters obtain better powerline noise removal results with increasing filter orders, their step responses are usually characterized by ringing and overshoot. In the same vein, depending on the filter order, the rate of attenuation can be low. Although the rate of attenuation in Chebyshev filters are higher than Butterworth filters, their step responses are marked by higher levels of ringing. Bessel filters do have significantly lower levels of ringing and overshoot, however their slower attenuation rates make it possible for powerline noise to leak into the signal.

Many signal processing algorithms have been proposed to solve the issue of powerline noise interference. Some of the notable procedures involve blind source separation. Empirical mode decomposition has been proposed as a potent approach for eliminating powerline noise (Blanco-Velasco, Weng & Barner, 2008; De-xiang, Xiao-pei & Xiao-jing, 2008; Li et al., 2012; Naji, Firoozabadi & Kahrizi, 2011; Nimunkar & Tompkins, 2007; Zhang & Zhou, 2013; Chang, 2010; Chang & Liu, 2011; Zivanovic & González-Izal, 2013). Independent component analysis has also been explored by many researchers as a potential approach for removing powerline noise (Xue et al., 2006; Iriarte et al., 2003; Castellanos & Makarov, 2006; Kuzilek et al., 2014; Chawla, 2011; Kuzilek, Kremen & Lhotska, 2014). A combination of empirical mode decomposition and independent component analysis has also been looked into as a potential approach for eliminating powerline noise (Mariyappa et al., 2015).

This paper proposes an algorithm which uses blind source separation and wavelet analysis to detect and remove powerline noise in biomedical signals. The approach is subsequently compared with a band-stop 4th order Butterworth filter, empirical mode decomposition, independent component analysis and a combination of empirical mode decomposition with independent component analysis. This unsupervised machine learning approach is fully automatable and void of the need to apply adaptive self-correction mechanisms.

The proposed approach is motivated by potency of the ensemble empirical mode decomposition algorithm in decomposing a signal into amplitude–frequency modulations—whose linear combination is results in the reconstruction of the signal. In this framework, these modulations are assumed to be a linearly mixed representation of source signals. Some of the modulations are then selected based on their frequency properties and un-mixed into their statistically independent sources via independent component analysis. The Morlet wavelet was used to describe the time-frequency properties of the decompositions and independent component analysis aided in extracting an optimal data-driven model of powerline noise. An inversion of the wavelet transform at 50/60 Hz of the powerline noise model was used to recover the powerline noise that exists within the signal. The result was subtracted from the original signal to obtain its denoised version.

Current algorithms that employ a combination of empirical mode decomposition and independent component analysis require the user to manually select the amplitude–frequency modulations and/or independent components of interest. An innovation of the proposed framework is that the signals of interest are automatically selected based on a pre-defined frequency threshold around their wavelet transformations. Another innovation of the proposed technique is that the inverse wavelet transform is applied on the powerline noise model—which was obtained via ensemble empirical mode decomposition and independent component analysis—to extract the 50/60 Hz component.

## Methods

### Pseudo-code for proposed approach


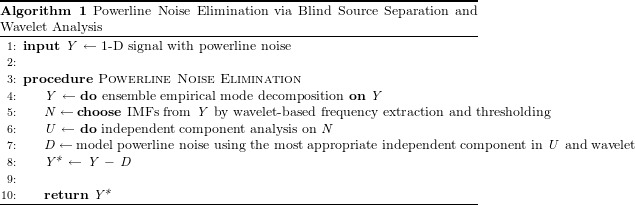


### Data source

The data used for the evaluation of this approach consisted of broadband neural activity from ensembles of hippocampal neurons, electrocardiographs and electroencephalogram signals (EEG signals).

• One set of data was obtained from the Collaborative Research in Computational Neuroscience initiative (Mizuseki et al., 2009). The recordings were made in the Cornu Ammonis 1 layer of the right dorsal hippocampus of Long Evans rats during open field foraging. This data was sampled at 20 kHz.
• Three randomly chosen sets of data with ECG signals from seemingly healthy volunteers were also employed (Goldberger et al., 2000; Garcia-Gonzalez et al., 2013; Citi, Poli & Cinel, 2010). These were sampled at 5 kHz.
• Six data sets of EEG signals from humans used for brain-computer interface applications were used (Goldberger et al., 2000). Three of them, which were previously used to elucidate the limitations of using event-related potentials for brain-computer interface applications, were sampled at 2,048 Hz (Citi, Poli & Cinel, 2010). The other half of the data sets were aimed at general purpose brain-computer interface design; they were sampled at 160 Hz (Schalk et al., 2008).

### Sliding time window

For the purpose of computational speed, a sliding window with no overlap was used.

### Decomposition of raw signal into amplitude-frequency modulations

#### Empirical mode decomposition

Suppose we want to eliminate a chronic sinusoidal *xHz* noise from a signal *y*(*t*) in a single channel. We can employ empirical mode decomposition (EMD) to split the time series signal into narrow-band amplitude–frequency sections (Huang et al., 1998; Wang et al., 2014). These sections, called intrinsic mode functions (IMFs), are obtained by an iterative sifting procedure. Initially, the local maxima and minima of the signal are detected and are connected by cubic splines to form the upper and lower envelopes respectively. The mean of the envelopes is then subtracted from the original signal to give rise to the first IMF. Subsequently, the first IMF is subtracted from the original signal to result in the residue. This process is repeated *k* times with the residue obtained at the end of each iteration serving as the input for the next. Ultimately, *k* IMFs and one residue will be obtained. The signal *y*(*t*) can be reconstructed by a summation of the IMFs *c_i_*(*t*) and the residue *r*(*t*):

(1) \begin{eqnarray*} \displaystyle y(t)=\sum _{i=1}^{k}{c}_{i}(t)+r(t).&&\displaystyle \end{eqnarray*}

The method by which the local extrema are detected is described in the Appendix (Ensemble empirical mode decomposition: detecting the local extrema).

#### Ensemble empirical mode decomposition

The ensemble empirical mode decomposition algorithm (EEMD) is an extension of EMD which is aimed at making the extraction of IMFs a robust process (Wu & Huang, 2009). In this algorithm, Gaussian noise of zero mean and a specified standard deviation is added to the input signal before EMD is applied. For the extraction of each IMF, the addition of Gaussian noise to the input is done over a specified number of ensembles and the mean extract in all the ensembles of putative IMFs is selected as the true IMF. This is a truly noise-assisted method of blind source separation because Gaussian noise forces the EMD algorithm to consider all options when sifting. With a high enough number of ensembles for each EEMD iteration, it can be inferred by central limit theorem that the mean of the ensembles is representative of the most likely IMF; thus, the fittest survive. The input signal for each ensemble can be summarized as follows:

(2) \begin{eqnarray*} \displaystyle G\sim \mathcal{N}(0,{\sigma }_{e}^{2});\qquad {\sigma }_{e}^{2}=[n l\ast \sigma \left(y(t)\right)]^{2};\qquad {e}_{{h}_{i n}}=y(t)+G&&\displaystyle \end{eqnarray*}

where *nl* is the desired inverse signal-to-noise ratio of the input signal and *e*_*h_in_*_ is the input signal for each ensemble *h*.

### Powerline noise detection

**Figure 1:**
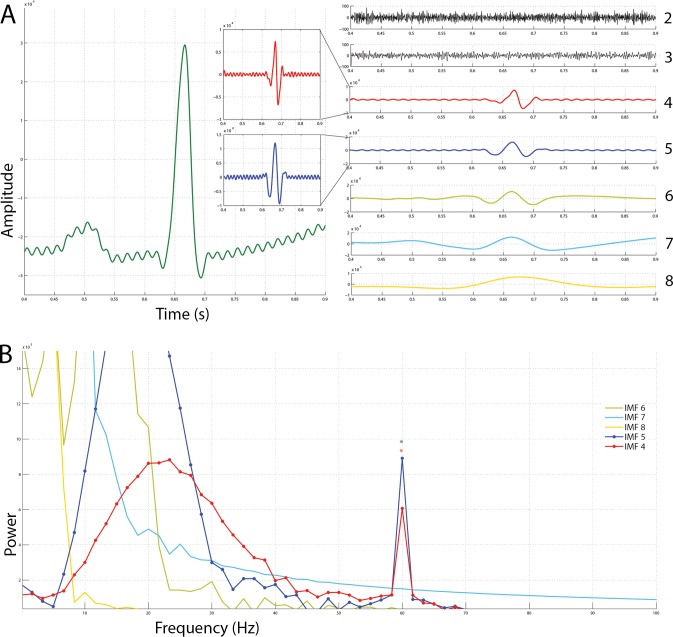
Ensemble empirical mode decomposition on an ECG signal with 60 Hz powerline noise added to it and the frequency spectra of the resulting intrinsic mode functions. (A) On the left is the original snippet of an ECG signal corrupted with powerline noise (green). Seven IMFs obtained via EEMD have been shown on the right. A constriction of the windows of IMF 4 and IMF 5 reveals that a chronic sinusoidal modulation exists within them. (B) The frequency spectra of the IMFs shown in A have been plotted. From this, it is plausible that the modulations seen in IMFs 4 and 5 are powerline noise at 60 Hz.

Due to the fact that IMFs obtained via EEMD have the attribute of being amplitude–frequency modulations (Fig. 1A), it is essential to un-mix the cocktail into their statistically independent sources. In principle, independent constituents of *y*(*t*) with specific frequencies—such as a chronic sinusoidal *xHz* noise—can be extracted with the aid of *c_i_*(*t*) and *r*(*t*). However, due to the high level of variability in the frequency spectrum for each IMF and the occasional overlap in their frequency spectra (Fig. 1B), selecting which IMFs will undergo independent component analysis will help reduce the probability of having noisy estimates of source components. Each IMF selected should be a putative powerline noise in accordance with the properties of its frequency.

#### IMF selection via wavelet analysis

A wavelet *φ*_(*a*,*b*)_(*t*) is square-integrable function that can be dilated (or constricted) along *a* and translated along *b*; it is written in the following form:

(3) \begin{eqnarray*} \displaystyle {\varphi }_{(a,b)}(t)=\frac{1}{\sqrt{a}}\left(\frac{t-b}{a}\right),\hspace{1em}\forall a\in {\mathfrak{R} }^{+}\wedge \forall b\in \mathfrak{R} .&&\displaystyle \end{eqnarray*}

For this framework, the real component of the consecutive Morlet wavelet was employed:

(4) \begin{eqnarray*} \displaystyle {\varphi }_{(f,j)}(t)=\left(\sqrt{1+{e}^{-{f}^{2}}-2{e}^{\frac{-3{f}^{2}}{4}}}\right)\left(\frac{1}{\sqrt{2 \pi }}{e}^{2 \pi i f}{e}^{\frac{-(t-j)^{2}}{2}}\right),&&\displaystyle \end{eqnarray*}

where *f* and *j* represent the center frequency (scale) and translation respectively. In order to describe the frequency properties of each independent component, a projection of the Morlet wavelet unto *c_i_*(*t*) = [*c*_1_(*t*), …, *c_k_*(*t*), *r*(*t*)]^*T*^ was used. This projection *p*(*F*, *i*) was by accomplished by finding the frequency *f* in the set of frequencies $F=\mathop{\{ {f}_{i}\} }\nolimits _{{f}_{i}=20}^{80}$ that maximizes a pseudo-convolution between *φ*_(*f*,*j*)_(*t*) and *c_i_*(*t*) and a summation of the pseudo-convolution values at all temporal locations *t*:

(5) \begin{eqnarray*} \displaystyle p(F,i)=\mathop{\mathop { \mathrm{argmax}}}\limits _{f\in F}\sum _{t_{k}}\left[\left\vert \left\vert \sum _{j_{k}}{\varphi }_{(f,j)}(t)\ast {c}_{i}(t)\right\vert -\frac{1}{{t}_{k}{f}_{k}}\sum _{t_{k}}\sum _{f_{k}}\left\vert \sum _{j_{k}}{\varphi }_{(f,j)}(t)\ast {c}_{i}(t)\right\vert \right\vert \right],&&\displaystyle \end{eqnarray*}

where *j_k_*, *t_k_* and *f_k_* are integers representing the *k^th^* translation, time and frequency evaluated respectively. A summation of the pseudo-convolution values at all temporal locations exposes the overall frequency morphology that describes a particular IMF. As mentioned previously, although each consecutive IMF exist within a lower frequency range, there is a likelihood of obtaining IMFs with partially overlapping frequency spectra (Fig. 1B). Thus, by choosing IMFs with dominant frequencies within a specified range, there is an appreciable degree of assurance that the *xHz* signal is in either of the chosen IMFs. Essentially, with Eq. (5), if *xHz* − *bHz* ≤ *p*(*F*, *i*) ≤ *xHz* + *bHz* (with *bHz* serving as a threshold for IMF selection), then ∃*i* such that *i* is the index that defines *c_i_*(*t*) as a putative *xHz* powerline noise. In the following subsections, any *c_i_*(*t*) such that *xHz* − *bHz* ≤ *p*(*F*, *i*) ≤ *xHz* + *bHz* is denoted as *n_q_*(*t*). This method is further elucidated in the Appendix (IMF selection).

#### Independent component analysis

Independent component analysis (ICA) is a data-driven approach to blind source separation of mixed input signals into their independent components. The premise of ICA is the assumption that each vectorial input signal *n_q_*(*t*) in *n* = [*n*_1_(*t*), …, *n_k_*(*t*)]^*T*^ is an observed signal which is a linearly mixed representation of its intrinsic source component *s_i_*(*t*) and components of statistically independent origin (Hyvärinen, Karhunen & Oja, 2004). This assumption infers the following:

(6) \begin{eqnarray*} \displaystyle n=A s,&&\displaystyle \end{eqnarray*}

where *A* is the mixing matrix and the source component *s* = [*s*_1_(*t*), …, *s_k_*(*t*)]. Inevitably, finding the un-mixing matrix *R* to obtain a matrix of output signals *u* which are estimates of *s_i_*(*t*) is the essence of ICA:

(7) \begin{eqnarray*} \displaystyle u=R n=R A s.&&\displaystyle \end{eqnarray*}

In this implementation of ICA, *R* was determined by an approximate simultaneous diagonalization of the third and fourth order cumulant tensors. Independent component analysis: finding R of the Appendix provides an explanation of how *R* was computed.

#### Powerline noise recognition

The powerline noise in *u* is recognized by extending the method outlined in Eq. (5) to search for the *u_i_* whose frequency properties resemble that of *xHz* powerline noise the most:

(8) \begin{eqnarray*} \displaystyle {d}_{c o m p}(t)=\mathop{\mathop { \mathrm{argmin}}}\limits _{{u}_{i}(t)}\left(\left\vert \left[\mathop{\mathop { \mathrm{argmax}}}\limits _{f\in F}\sum _{t_{k}}\left(\left\vert \left\vert \sum _{j_{k}}{\varphi }_{(f,j)}(t)\ast {u}_{i}(t)\right\vert -\frac{1}{{t}_{k}{f}_{k}}\sum _{t_{k}}\sum _{f_{k}}\left\vert \sum _{j_{k}}{\varphi }_{(f,j)}(t)\ast {u}_{i}(t)\right\vert \right\vert \right)\right]-x \hspace{0.167em} \right\vert \right),&&\displaystyle \nonumber\\ \displaystyle &&\displaystyle \end{eqnarray*}

where *x* represents the expected frequency of the powerline noise (50 Hz or 60 Hz). In essence, the *u_i_*(*t*) that satisfies Eq. (8) is the most appropriate approximation of powerline noise. The motivation behind this approach has been explicated in Powerline noise recognition of the Appendix.

### Signal reconstruction

Powerline noise is modeled by inverting a convolution between *d_comp_*(*t*) and the real portion of the Morlet wavelet, and normalizing the root-mean-square amplitude—via learning and estimating the least square distance aggregate—to compensate for the loss of amplitude information due to ICA (Appendix, Signal reconstruction):

(9) \begin{eqnarray*} \displaystyle d(t)&=&\displaystyle A{C}^{-1}\frac{\lim _{T\rightarrow \infty }\sqrt{{T}^{-1}\int \nolimits _{0}^{T}{\left[\sum _{j_{k}}{\varphi }_{(x\in S,j)}(t)\ast y(t)\right]}^{2}d t}}{\lim _{T\rightarrow \infty }\sqrt{{T}^{-1}\int \nolimits _{0}^{T}{\left[\sum _{j_{k}}{\varphi }_{(x\in S,j)}(t)\ast {d}_{c o m p}(t)\right]}^{2}d t}}\nonumber\\ \displaystyle &&\displaystyle \times \, \left[\sum _{j}{\varphi }_{(x\in S,j)}(t)\left(\sum _{j_{k}}{\varphi }_{(x\in S,j)}(t)\ast {d}_{c o m p}(t)\right)\right],\nonumber\\ \displaystyle &&\displaystyle \text{where }S=\{ 50,60\} \wedge C={\left(\sum _{t}\left\vert {\varphi }_{(x\in S,j)}(t)\right\vert \right)}^{2}\wedge A\in \mathfrak{R} . \end{eqnarray*}

In the same light as Eq. (1), the denoised signal $\widehat{y(t)}$ can be reconstructed in the following manner:

(10) \begin{eqnarray*} \displaystyle \widehat{y(t)}=\sum _{i=1}^{k}{c}_{i}(t)+r(t)-d(t)=y(t)-d(t).&&\displaystyle \end{eqnarray*}

## Results and Discussion

In this section, properties of the proposed algorithm are explored. The performance of the proposed denoising algorithm is evaluated in the presence of artificial and natural powerline noise. Subsequently, the performance with varying SNRs is compared with that of a band-stop 4th order Butterworth filter, empirical mode decomposition, independent component analysis and the combination of empirical mode decomposition with independent component analysis.

### Noise extraction and signal reconstruction

#### Characteristics of the proposed approach

**Figure 2:**
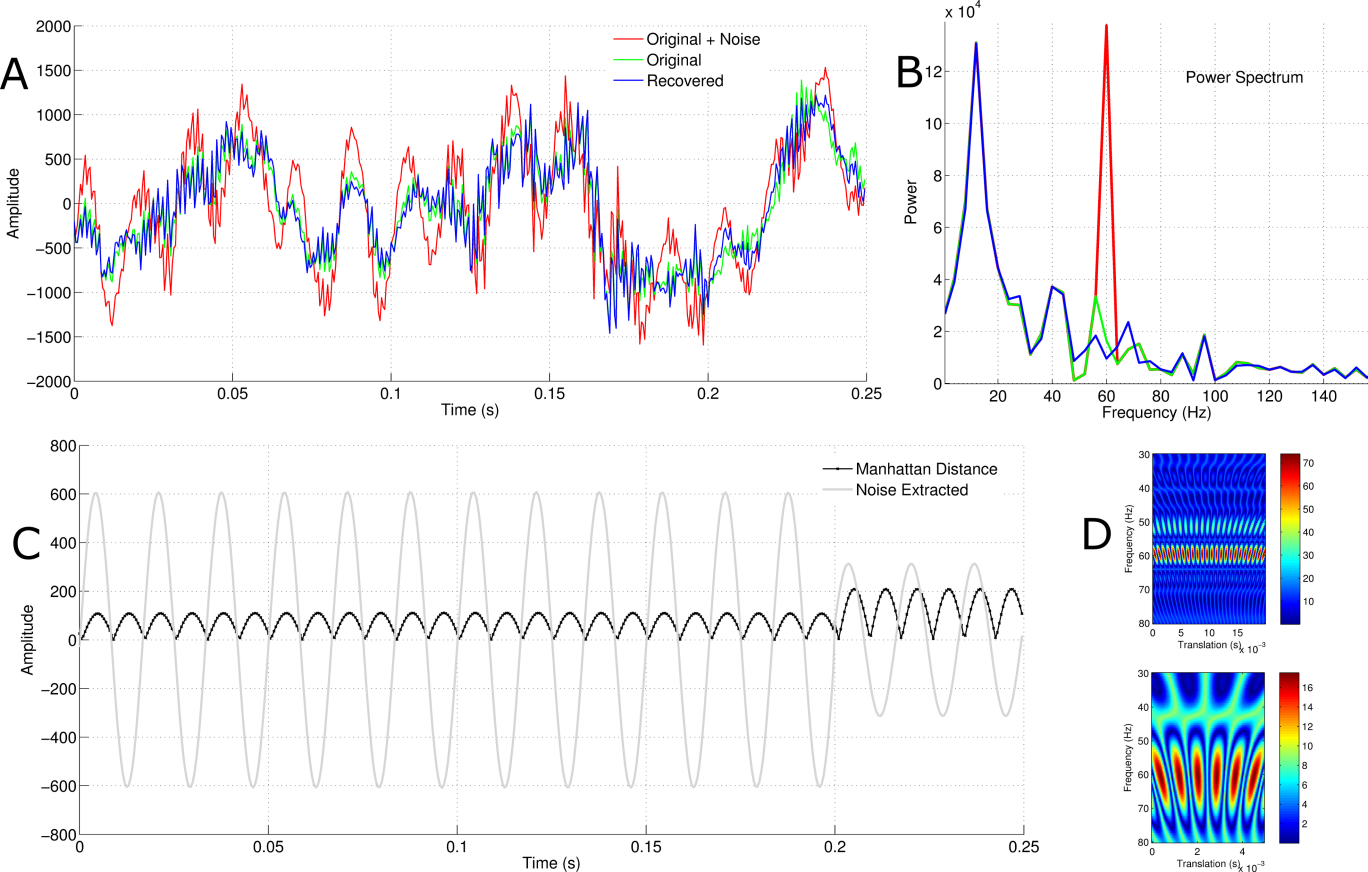
Independent component selection, signal reconstruction and the effect of time window. (A) The blue, green and red signals are the recovered, original and noised signals respectively. (B) The blue, green and red signals are the power spectrum for the recovered, original and noised signals respectively. (C) The grey trace is the AC noise extracted and the black trace is the Manhattan distance between the original signal and the recovered signal. (D) The upper image is the pseudo-convolution of the selected independent component for the first 200 ms of the data. The lower represents that of the final 50 ms. Note that they both peak at circa 60 Hz.

To demonstrate the viability of the process of powerline noise removal, artificial 60 Hz noise was added to local field potentials (SNR = 4.2816dB; amplitude = 700) and was subsequently reconstructed. A 200 ms time window (without overlap) was used to denoise 250 ms of the mentioned neural data. As shown in Figs. 2A and 2C, the procedure was able to recover a sufficient amount of the original signal with little Manhattan distances. The morphology of the Manhattan distance suggests that the procedure extracted the appropriate frequency, but obtained relatively erroneous amplitude information. As shown in Figs. 2B and 2D, the pseudo-convolution procedure for extraction of powerline noise chose the 60 Hz noise—which is the right frequency for the noise model in this context. Since the pseudo-convolution between the extracted alternating current noise peaks at about 60 Hz on all translations, it implies that the summation of the pseudo-convolution values on every translation should result in a function that also peaks at about 60 Hz. Thus, whichever independent component that has its summed pseudo-convolution peak closest to 60 Hz is indeed the desired powerline noise. Although the final round of blind-source separation in this algorithm requires prior knowledge about the statistical properties of the source data being approximated, it is plausible that this constraint is not a hindrance but, instead, facilitates the process. With the assumption of statistical independence, at least one of the resulting approximations of the source data is forced to look undeniably unique. Signals of this form are usually some fluctuations that are chronically present in the mixed data. For this reason, ICA effectively serves as a helper for the extraction and identification of the desired powerline noise to be removed. The Manhattan distance between the original signal and the denoised signal increased during the final 50 ms of the neural data. This suggests the approach does not provide good results with very small time windows. Essentially, the best approach will be to run the algorithm without a time window. In conclusion, it is most appropriate for offline signal analysis.

#### Learning the noise amplitude

**Figure 3:**
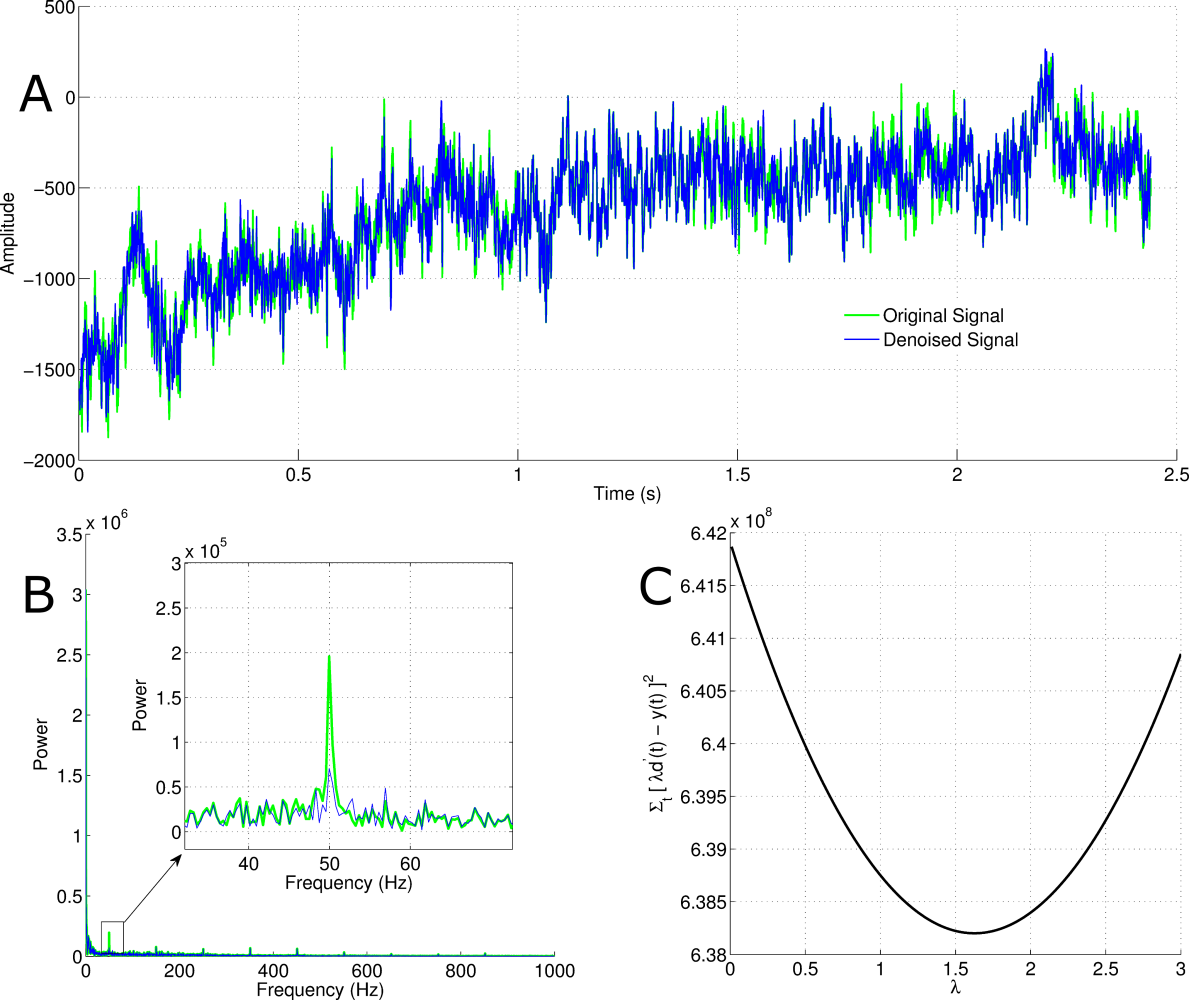
Learning the amplitude of powerline noise in a natural context. (A) The green trace is the original signal and the blue trace is the reconstructed signal after removing natural 50 Hz AC noise. (B) The green trace represents the power spectrum for the original signal and the blue one represents that of the reconstructed signal. (C) This shows the process by which the noise amplitude is learned by the algorithm.

In accordance with the fact that the problem of deconvolution is ill-posed, the solutions are not unique; this issue is tackled by learning the appropriate amplitude for the noise model. To exhibit the amplitude learning procedure, an EEG with natural 50 Hz noise was denoised using the proposed framework. As presented in Fig. 3C, the learning procedure aimed at minimizing the cost function has a unique solution. This indicates that there is some level of consistency in the process. In view of the fact that there is virtually no ground truth with natural AC noise, the power spectra of the original signal and the denoised signal were compared. The traces in Fig. 3A show that there is little difference between the original signal and the recovered signal. However, Fig. 3B reveals that this manifestation is primarily due to the low amplitude of the 50 Hz AC noise. Although the amplitude is very small, the algorithm was able to extinguish an appreciable amount of powerline noise. The same figure indicates that if the amplitude of the AC noise is small enough, it might go unnoticed after Fourier transformation. Further, in order to obtain an accurate view of how a biomedical signal behaves under various frequency specifications, it is better to employ the wavelet transform (which is a convolution between a wavelet and a signal) rather than the Fourier transform. This is due to the fact that the Fourier transform assumes the signal being transformed is of a sinusoidal origin (Bloomfield, 2004), which is not true in most cases. Although the short-term Fourier transform is used to avoid this pitfall, the wavelet transform (which handles frequencies in a logarithmic fashion) adapts better to highly variable signals (Daubechies, 1990). The suggested algorithm employed a transformation analytically similar to the wavelet transform. By the same token, the frequency properties revealed by a summation of the pseudo-convolution between the real component of the Morlet wavelet and the signals aided in reliably in extracting powerline noise; this, in turn, provided a solid model foundation for learning the most appropriate amplitude.

### Artificial corruption of biomedical signals with powerline noise

#### Neural signals

**Figure 4:**
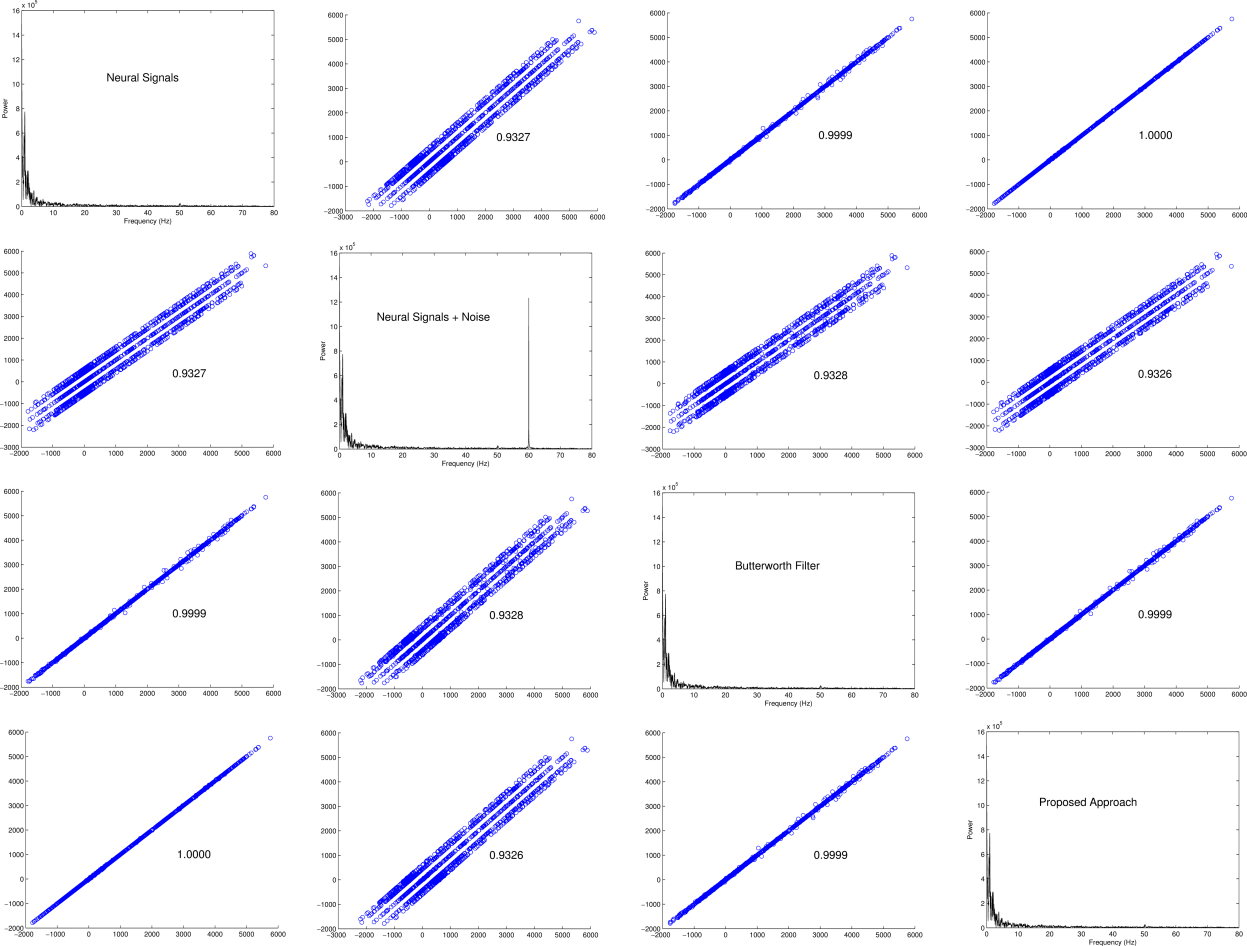
Scatterplot matrix of results obtained with neural signals. The off-diagonal elements are scatterplots of the signal amplitudes and the diagonal elements are Fourier transformations of the signals. For each of the off-diagonal elements, the abscissa is the label of nearest diagonal element above or below and the ordinate is the label of the nearest diagonal element on its side. For example, on the first row and second column the amplitudes of neural signals without noise is compared with the same version adulterated with powerline noise using a scatterplot. On the first row and third column, neural signals without noise is compared with a reconstructed version using a 4th order Butterworth filter. On the first row and fourth column, the results obtained via the proposed approach is compared with the original neural signals. This convention is used throughout the figure.

Sub-cortical local field potentials and extra-cranial EEGs were resampled to have equal sampling rate, concatenated and adulterated with artificial powerline noise. A band-stop 4th order Butterworth filter was used to filter out signals between 59.5 Hz and 60.5 Hz, and the proposed framework was also applied to the adulterated signal. The introduction of powerline noise introduced a slight amplitude decorrelation between the original signal and the adulterated signal. The aim of Fig. 4 was to characterize the level of re-correlation after removing the powerline noise using the proposed approach and the mentioned infinite impulse response filter (IIR filter). It was noted that although the frequency spectra indicated that both methods expelled powerline noise, their correlations differed minutely. The proposed approach recovered the signals with a slightly higher correlation than the named IIR filter (proposed = 1.0000, Butterworth filter = 0.9999).

**Figure 5:**
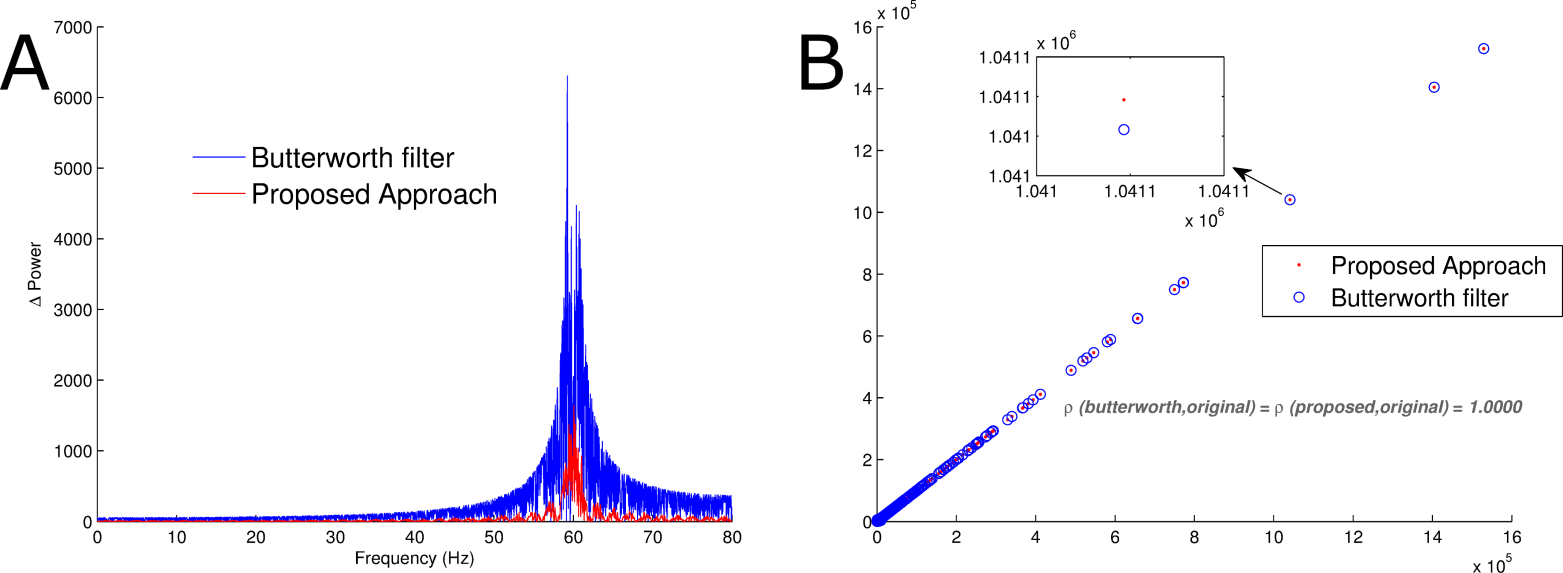
Comparison of power spectra. (A) The red trace represents the Manhattan distance between the Fourier transformation of the proposed reconstruction procedure and the original signal without artificial noise. Similarly, the blue trace is the Manhattan distance between the Fourier transformation of the signal obtained via band-stop Butterworth filtering and the original signal without artificial noise. (B) The red trace is a scatterplot of the Fourier transform of the original signal and the reconstructed signal using the proposed approach. Likewise, the blue trace represents a scatterplot of the Fourier transform of the original signal and the reconstructed signal using a band-stop Butterworth filter. Pearson’s correlation coefficient for both were 1.0000.

In order to understand the subtle differences between the proposed method and the mentioned IIR filter, the Manhattan distance between the Fourier transformation of the original signal which had no powerline noise and the denoised signal using both approaches were evaluated (Fig. 5). It is shown that the difference between the power spectrum of the original signal and the one reconstructed using the proposed approach was smaller than that of the band-stop Butterworth filter. This was further validated via a Wilcoxon rank-sum test; the *p*-value obtained by comparing the original power spectrum with that of the one reconstructed using the proposed approach was 0.9903, while that of the band-stop Butterworth filter was 0.7627. In spite of the fact that there was no statistically significant difference between the reconstructed power spectra and that of the original, there was a statistically significant difference between the power spectrum of the signals reconstructed using the suggested algorithm and that of the band-stop IIR filter (*p*-value < 0.0001). On a grand scale, the power spectra of the denoised signals were highly correlated. This is an indication that there was a relatively similar response at the powerline noise frequency for neural signals.

#### Electrocardiogram signals

**Figure 6:**
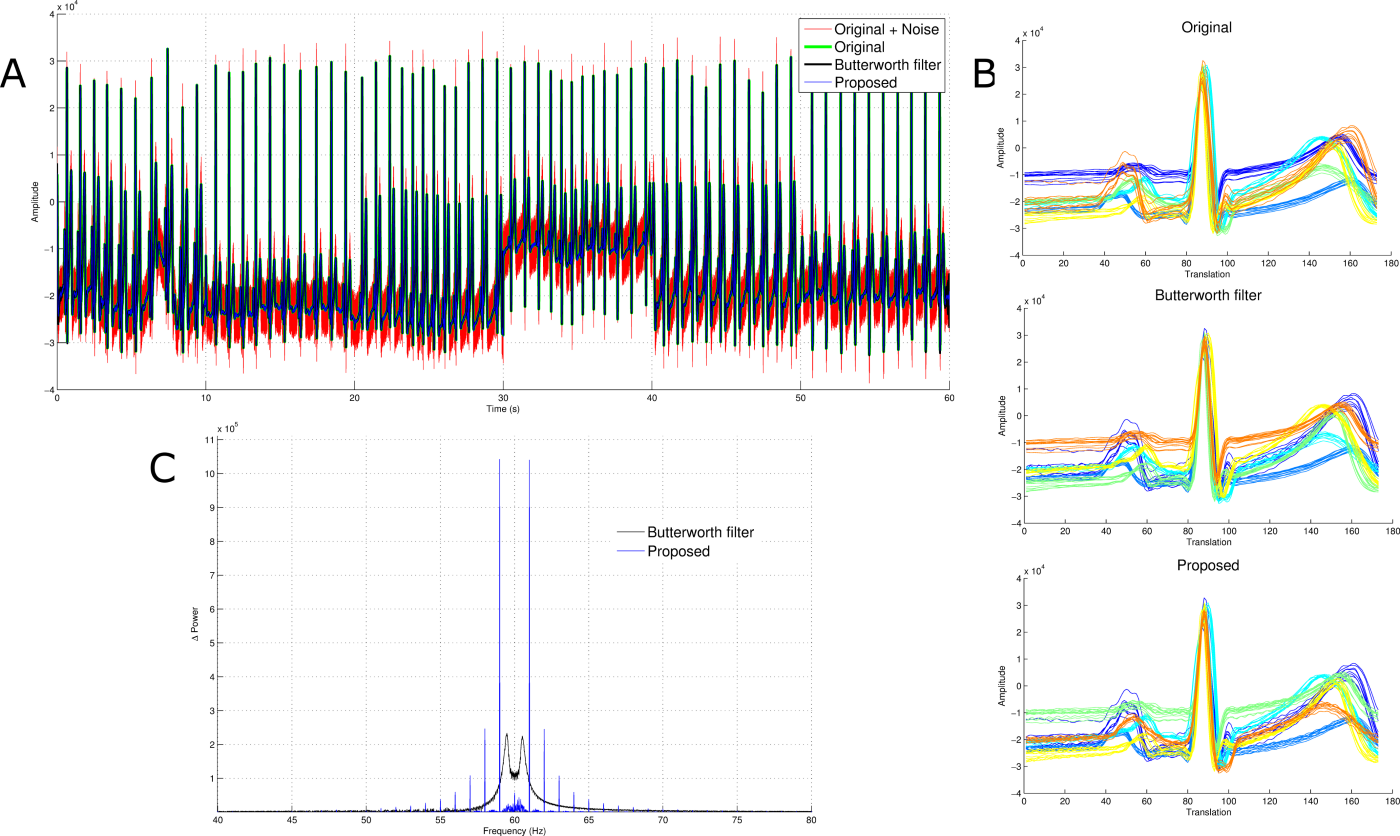
Comparison of power spectra and waveforms. (A) The red, green, black and blue traces are the adulterated signal, original signal, signal obtained after Butterworth filter and signal obtained using the proposed denoising framework. (B) The waveforms detected via k-means without dimensionality reduction. (C) The blue signal is the Manhattan distance between the Fourier transform of the results from the proposed approach and the original signal. Similarly, the black trace is the Manhattan distance between the Fourier transform of the signal obtained via Butterworth filtering and the original signal.

Electrocardiogram signals without powerline noise were corrupted with artificial 60 Hz powerline noise. Thereafter, they were reconstructed via band-stop Butterworth filtering and the proposed method. The results obtained were almost identical in the time series (Fig. 6A), however there was a substantial difference circa 60 Hz. Similar to the results obtained with neural signals, the power of the extract using the proposed method at 60 Hz was lower than that of the Butterworth filter. One of the many properties of ECG signals sought after is the waveform. In accordance with the knowledge that the ECG signal used was a concatenation of three data sets with two electrode recordings each, it is expected that at least six different clusters ECG waveforms can be detected. The waveforms were detected via simple thresholding and were partitioned via the k-means algorithm. Naturally, clustering such high dimensional data requires dimensionality reduction via principal component analysis or laplacian eigenmaps. This clustering process was done without dimensionality reduction in order to accurately compare the effect of the proposed approach and that of the Butterworth filter on the reconstructed signal. As shown in Fig. 6B, although the labels of the classes were different, their elements were not. This further proves that the relative temporal morphology of each reconstructed signal is preserved.

### Natural powerline noise removal

**Figure 7:**
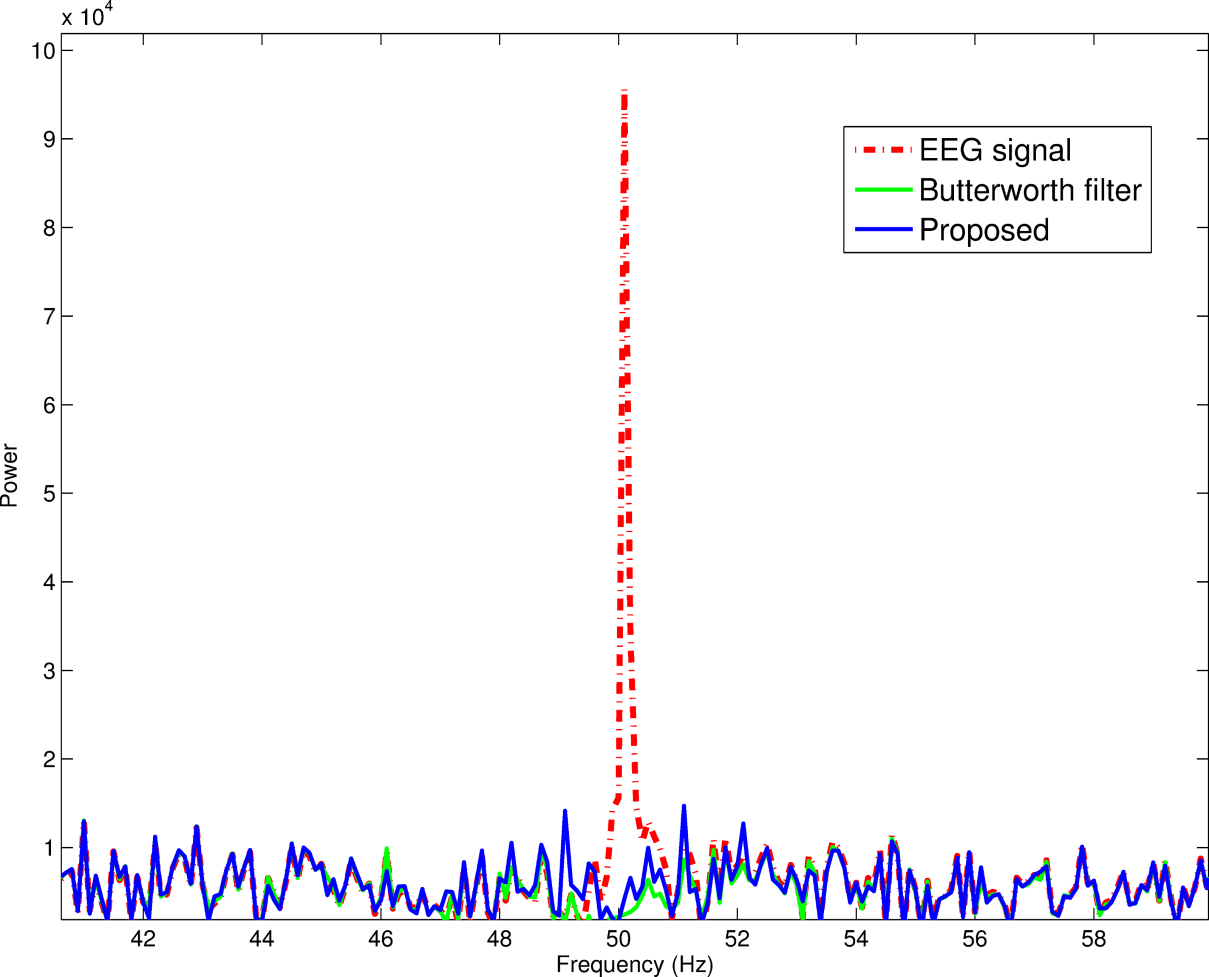
Powerline noise removal in a natural setting. The proposed method and the stop-band Butterworth filter extinguished natural powerline noise in a comparable fashion.

To evaluate the utility of the proposed method in a natural setting, an EEG data set with 50 Hz powerline noise was used. As shown in Fig. 7, the proposed framework and the band-stop 4th order Butterworth filter both removed the powerline noise. This further proves the potency of the proposed method.

### Comparison with other approaches

**Figure 8:**
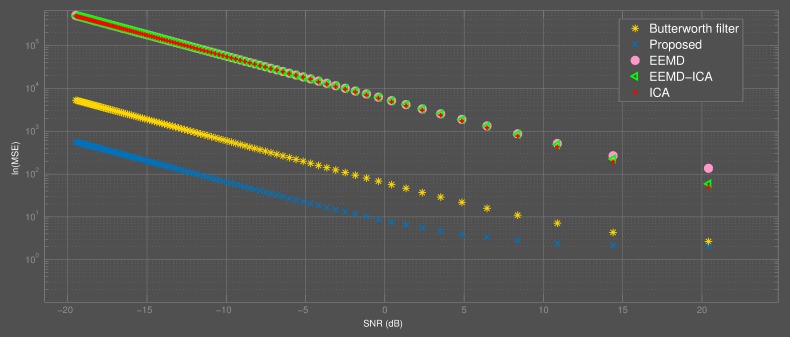
Comparison of the proposed method with other approaches. The proposed approach performed better than others at all SNRs considered.

The performance of the proposed approach was compared with that of a 4th order band-stop Butterworth filter, EEMD, ICA and a combination of EEMD and ICA (EEMD-ICA). The log-mean squared error between the original signal and the signal recovered after eliminating powerline noise was used as a parameter to compare the performance of the approaches. With Fig. 8, it is shown that the proposed approach performed better than the approaches mentioned previously. It can be noted that EEMD, ICA and EEMD-ICA had comparable results from a broad perspective; however ICA performed slightly better under high SNRs. From this, it is plausible that the reason why EEMD-ICA had lower log-mean squared errors at higher SNRs than EEMD alone was due to the effect of ICA. The mentioned infinite impulse response filter—which is a state-of-the-art technique—proved to be better at eliminating powerline noise than all the mentioned approaches except the proposed framework. In contrast with the other techniques, the change in performance (from a log-mean squared error point of view) plateaued rather quickly at SNRs greater than 0dB; however, it still maintained the highest performance across all SNRs evaluated. This is a strong indication that the proposed approach works relatively better at low SNRs.

## Conclusion

A framework for the elimination of powerline noise in biomedical signals has been introduced. This adaptive method does not make assumptions on linearity, stationarity nor time-invariance and is virtually void of the need for self-correction mechanisms. Pattern recognition of the extracted features by wavelet analysis provides an enhancement to this procedure by making it fully automatable and completely unsupervised. It is worth noting that this approach is better suited for offline analysis due to its sensitivity to the time window.

## Appendix. Blind source separation

### Ensemble empirical mode decomposition: detecting the local extrema

The local maxima *α_max_* and minima *α_min_* are found by the following:

(11) \begin{eqnarray*} \displaystyle {\alpha }_{m a x}=({y}_{m a x},x(t));\qquad {\alpha }_{m i n}=({y}_{m i n},x(t)),&&\displaystyle \end{eqnarray*}

where *y_max_* = *y*(*t*), if *y*(*t*) > *y*(*t* − 1)∧*y*(*t*) > *y*(*t* + 1), *y_min_* = *y*(*t*), if *y*(*t*) < *y*(*t* − 1)∧*y*(*t*) < *y*(*t* + 1) and *x*(*t*) = *t*. As noted previously, *α_max_* and *α_min_* serve as the nodes for cubic spline interpolation to extract the upper and lower envelopes respectively. Cubic splines connect nodes such that the first and second derivatives of the interpolation between each node are continuous. The cubic spline function used to connect each node in an interval (*y_n_*, *y*_*n*+1_) is as follows:

(12) \begin{eqnarray*} \displaystyle f=A{f}_{n}+B{f}_{n+1}+C{f}_{n}^{{\prime\prime}}+D{f}_{n+1}^{{\prime\prime}},&&\displaystyle \end{eqnarray*}

where $A\equiv \frac{{y}_{n+1}-y}{{y}_{n+1}-{y}_{n}}$, *B* ≡ 1 − *A*, $C\equiv \frac{1}{6}({A}^{3}-A)({y}_{n+1}-{y}_{n})^{2}$ and $D\equiv \frac{1}{6}({B}^{3}-B)({y}_{n+1}-{y}_{n})^{2}$.

### Independent component analysis: finding R

In accordance with the initial assumption that ∀*s_i_*(*t*) are statistically independent of each other, *u* can be described by its cumulants ${C}_{\ldots }^{u}$ to reveal its statistical properties. As an extension, the off-diagonal elements erode and the cumulant tensors of all orders are diagonal if and only if ∀*u_i_*(*t*) are independent of each other. Since the first order cumulant tensor does not have off-diagonal elements and the second order cumulant tensor can be diagonalized by whitening *n*, *u* can be represented as follows:

(13) \begin{eqnarray*} \displaystyle u=Q W n=R n,&&\displaystyle \end{eqnarray*}

where *W* is the whitening matrix and *Q* is an orthogonal transformation matrix that approximately diagonalizes the third and fourth order cumulant tensors simultaneously. Consequentially, it is integral to employ an optimization framework to find the orthogonal matrix *Q* that maximizes the following contrast function:

(14) \begin{eqnarray*} \displaystyle {\Psi }_{34}(Q)=\frac{1}{3 !}\sum _{\alpha }{\left(\sum _{\beta \gamma \delta }{Q}_{\alpha \beta }{Q}_{\alpha \gamma }{Q}_{\alpha \delta }{C}_{\beta \gamma \delta }^{(W n)}\right)}^{2}+\frac{1}{4 !}\sum _{\alpha }{\left(\sum _{\beta \gamma \delta \epsilon }{Q}_{\alpha \beta }{Q}_{\alpha \gamma }{Q}_{\alpha \delta }{Q}_{\alpha \epsilon }{C}_{\beta \gamma \delta \epsilon }^{(W n)}\right)}^{2}.&&\displaystyle \end{eqnarray*}

Without loss of generality, the Givens rotation for two components *μ* and *ξ* with rotation angle *θ* and Kronecker delta *δ*_*αβ*_ can be evaluated in the following manner:

(15) \begin{eqnarray*} \displaystyle {Q}_{\alpha \beta }^{\mu \xi }=\left\{\begin{array}{ll} \displaystyle \cos (\theta ),\hspace{1em}&\displaystyle \text{for }(\alpha ,\beta )\in \{ (\mu ,\mu ),(\xi ,\xi )\} \\ \displaystyle -\sin (\theta ),\hspace{1em}&\displaystyle \text{for }(\alpha ,\beta )\in \{ (\mu ,\xi )\} \\ \displaystyle \sin (\theta ),\hspace{1em}&\displaystyle \text{for }(\alpha ,\beta )\in \{ (\xi ,\mu )\} \\ \displaystyle {\delta }_{\alpha \beta },\hspace{1em}&\displaystyle \text{otherwise}. \end{array}\right.\hspace{2.77695pt}\Longrightarrow \hspace{2.77695pt}{Q}^{\mu \xi }=\left[\begin{array}{cc} \displaystyle \cos (\theta )&\displaystyle \sin (\theta )\\ \displaystyle -\sin (\theta )&\displaystyle \cos (\theta ) \end{array}\right].&&\displaystyle \end{eqnarray*}

This extends the optimization procedure to find *θ* which maximizes an equivalent of the contrast function laid out in Eq. (14):

(16) \begin{eqnarray*} \displaystyle \begin{array}{l} \displaystyle {\Psi }_{34}(\theta )={\Psi }_{3}(\theta )+{\Psi }_{4}(\theta );\\ \displaystyle {\Psi }_{n}(\theta )\coloneq \frac{1}{n !}\sum _{i=0}^{n}{d}_{n i}(\cos (\theta )^{(2 n-i)}\sin (\theta )^{i})+\frac{1}{n !}\sum _{i=0}^{n}{d}_{n i}(\cos (\theta )^{(i)}(-\sin (\theta ))^{(2 n-i)}), \end{array}&&\displaystyle \end{eqnarray*}

where *d_ni_* represents constants that depend on the cumulants prior to rotation; these constants have been explicated by Blaschke & Wiskott (2004). From this decomposition, it is evident that a reversal of this unique *θ* gives rise to the coveted orthogonal transformation matrix *Q* and, as a consequence, provides estimates of *s_i_*(*t*).

## Powerline noise detection

### IMF selection

The wavelet transform *W*_*φ*_*c_i_*_(*f*,*j*)_ is a convolution between a wavelet *φ*_(*f*,*j*)_ and a function *c_i_*(*t*):

(17) \begin{eqnarray*} \displaystyle {{W}_{\varphi }{c}_{i}}_{(f,j)}=\int \nolimits _{-\infty }^{\infty }{\varphi }_{(f,j)}{c}_{i}(t)d t.&&\displaystyle \end{eqnarray*}

Depending on the wavelet used, this transformation has the potential to provide an accurate description of the frequency properties of the signal. In a similar fashion, due to the fact that powerline noise has a similar morphology to that of the Morlet wavelet, the following term in Eq. (5) (with *j* ≡ *t*) is used to obtain the temporal frequency spectrum:

(18) \begin{eqnarray*} \displaystyle {T}_{(f,t)}=\left\vert \sum _{j_{k}}{\varphi }_{(f,j)}(t)\ast {c}_{i}(t)\right\vert .&&\displaystyle \end{eqnarray*}

Eq. (18) can be represented in the following matrix form:

(19) \begin{eqnarray*} \displaystyle T=\left[\begin{array}{ccc} \displaystyle \left\vert \sum _{j_{k}}{\varphi }_{({f}_{1},j)}(t)\ast {c}_{1}({t}_{1})\right\vert &\displaystyle \cdots \hspace{0.167em} &\displaystyle \left\vert \sum _{j_{k}}{\varphi }_{({f}_{1},j)}(t)\ast {c}_{1}({t}_{k})\right\vert \\ \displaystyle \vdots &\displaystyle \ddots &\displaystyle \vdots \\ \displaystyle \left\vert \sum _{j_{k}}{\varphi }_{({f}_{k},j)}(t)\ast {c}_{i}({t}_{1})\right\vert &\displaystyle \cdots \hspace{0.167em} &\displaystyle \left\vert \sum _{j_{k}}{\varphi }_{({f}_{k},j)}(t)\ast {c}_{i}({t}_{k})\right\vert \end{array}\right].&&\displaystyle \end{eqnarray*}

Subsequently, the mean of *T* is subtracted from *T* and the absolute value of the result is computed:

(20) \begin{eqnarray*} \displaystyle \begin{array}{l} \displaystyle \mu =\frac{1}{{t}_{k}{f}_{k}}\sum _{t_{k}}\sum _{f_{k}}T=\frac{1}{{t}_{k}{f}_{k}}\sum _{t_{k}}\sum _{f_{k}}\left\vert \sum _{j_{k}}{\varphi }_{(f,j)}(t)\ast {c}_{i}(t)\right\vert ,\\ \displaystyle \begin{array}{rcl} \displaystyle N&\displaystyle =&\displaystyle \left\vert T-\mu \right\vert =\left\vert T-\frac{1}{{t}_{k}{f}_{k}}\sum _{t_{k}}\sum _{f_{k}}T\right\vert \\ \displaystyle &\displaystyle =&\displaystyle \left\vert \left\vert \sum _{j_{k}}{\varphi }_{(f,j)}(t)\ast {c}_{i}(t)\right\vert -\frac{1}{{t}_{k}{f}_{k}}\sum _{t_{k}}\sum _{f_{k}}\left\vert \sum _{j_{k}}{\varphi }_{(f,j)}(t)\ast {c}_{i}(t)\right\vert \right\vert . \end{array} \end{array}&&\displaystyle \end{eqnarray*}

This can also be represented in the matrix form as follows:

(21) \begin{eqnarray*} \displaystyle N=\left[\begin{array}{ccc} \displaystyle \left\vert {T}_{({f}_{1},j)}-\frac{1}{{t}_{k}{f}_{k}}\sum _{t_{k}}\sum _{f_{k}}\left\vert \sum _{j_{k}}{\varphi }_{(f,j)}(t)\ast {c}_{i}({t}_{1})\right\vert \right\vert &\displaystyle \cdots \hspace{0.167em} &\displaystyle \left\vert {T}_{({f}_{1},j)}-\frac{1}{{t}_{k}{f}_{k}}\sum _{t_{k}}\sum _{f_{k}}\left\vert \sum _{j_{k}}{\varphi }_{(f,j)}(t)\ast {c}_{i}({t}_{k})\right\vert \right\vert \\ \displaystyle \vdots &\displaystyle \ddots &\displaystyle \vdots \\ \displaystyle \left\vert {T}_{({f}_{k},j)}-\frac{1}{{t}_{k}{f}_{k}}\sum _{t_{k}}\sum _{f_{k}}\left\vert \sum _{j_{k}}{\varphi }_{(f,j)}(t)\ast {c}_{i}({t}_{1})\right\vert \right\vert &\displaystyle \cdots \hspace{0.167em} &\displaystyle \left\vert {T}_{({f}_{k},j)}-\frac{1}{{t}_{k}{f}_{k}}\sum _{t_{k}}\sum _{f_{k}}\left\vert \sum _{j_{k}}{\varphi }_{(f,j)}(t)\ast {c}_{i}({t}_{k})\right\vert \right\vert \end{array}\right].&&\displaystyle \end{eqnarray*}

By summing this pseudo-convolution at all temporal locations, a column vector is obtained:

(22) \begin{eqnarray*} \displaystyle \sum _{t_{k}}N&=&\displaystyle \left[\begin{array}{c} \displaystyle \sum _{t_{k}}\left\vert {T}_{({f}_{1},j)}-\frac{1}{{t}_{k}{f}_{k}}\sum _{t_{k}}\sum _{f_{k}}\left\vert \sum _{j_{k}}{\varphi }_{(f,j)}(t)\ast {c}_{i}({t}_{k})\right\vert \right\vert \\ \displaystyle \vdots \\ \displaystyle \sum _{t_{k}}\left\vert {T}_{({f}_{k},j)}-\frac{1}{{t}_{k}{f}_{k}}\sum _{t_{k}}\sum _{f_{k}}\left\vert \sum _{j_{k}}{\varphi }_{(f,j)}(t)\ast {c}_{i}({t}_{k})\right\vert \right\vert \end{array}\right]\nonumber\\ \displaystyle &=&\displaystyle \sum _{t_{k}}\left(\left\vert \left\vert \sum _{j_{k}}{\varphi }_{(f,j)}(t)\ast {c}_{i}(t)\right\vert -\frac{1}{{t}_{k}{f}_{k}}\sum _{t_{k}}\sum _{f_{k}}\left\vert \sum _{j_{k}}{\varphi }_{(f,j)}(t)\ast {c}_{i}(t)\right\vert \right\vert \right). \end{eqnarray*}

Thus, the frequency *f* which best describes the IMF, for the purpose of powerline noise detection, can be found in the manner laid out in Eq. (5):

(23) \begin{eqnarray*} \displaystyle p(F,i)=\mathop{\mathop { \mathrm{argmax}}}\limits _{f\in F}\sum _{t_{k}}\left(\left\vert \left\vert \sum _{j_{k}}{\varphi }_{(f,j)}(t)\ast {c}_{i}(t)\right\vert -\frac{1}{{t}_{k}{f}_{k}}\sum _{t_{k}}\sum _{f_{k}}\left\vert \sum _{j_{k}}{\varphi }_{(f,j)}(t)\ast {c}_{i}(t)\right\vert \right\vert \right),&&\displaystyle \end{eqnarray*}

where any IMF whose with index *i* such that *xHz* − *bHz* ≤ *p*(*F*, *i*) ≤ *xHz* + *bHz* is a probable powerline noise. The threshold *b* is used to find which IMFs are close to the *xHz* powerline noise in order to avoid losing putative powerline noise in the extraction process.

## Powerline model and signal reconstruction

### Powerline noise recognition

After ICA, an extension of a variation of Eq. (23) can be used to extract the independent component whose frequency property is most akin to that of powerline noise. Assume the independent components extracted are of the form *u_i_*(*t*). By subtracting the *xHz*, it can be inferred that the independent component which resembles powerline noise the most is the one whose maximum frequency is closest to zero. Thus, the *u_i_*(*t*) which minimizes the absolute value of the mentioned operation is the most suitable model for powerline noise:

(24) \begin{eqnarray*} \displaystyle {d}_{c o m p}(t)&=&\displaystyle \mathop{\mathop { \mathrm{argmin}}}\limits _{{u}_{i}(t)}\left(\left[\begin{array}{c} \displaystyle \left\vert \left[\mathop{\mathop { \mathrm{argmax}}}\limits _{f\in F}\sum _{t_{k}}\left(\left\vert \left\vert \sum _{j_{k}}{\varphi }_{(f,j)}(t)\ast {u}_{1}(t)\right\vert -\frac{1}{{t}_{k}{f}_{k}}\sum _{t_{k}}\sum _{f_{k}}\left\vert \sum _{j_{k}}{\varphi }_{(f,j)}(t)\ast {u}_{1}(t)\right\vert \right\vert \right)\right]-x\right\vert \\ \displaystyle \vdots \\ \displaystyle \left\vert \left[\mathop{\mathop { \mathrm{argmax}}}\limits _{f\in F}\sum _{t_{k}}\left(\left\vert \left\vert \sum _{j_{k}}{\varphi }_{(f,j)}(t)\ast {u}_{i}(t)\right\vert -\frac{1}{{t}_{k}{f}_{k}}\sum _{t_{k}}\sum _{f_{k}}\left\vert \sum _{j_{k}}{\varphi }_{(f,j)}(t)\ast {u}_{i}(t)\right\vert \right\vert \right)\right]-x\right\vert \end{array}\right]\right)\nonumber\\ \displaystyle &=&\displaystyle \mathop{\mathop { \mathrm{argmin}}}\limits _{{u}_{i}(t)}\left(\left\vert \left[\mathop{\mathop { \mathrm{argmax}}}\limits _{f\in F}\sum _{t_{k}}\left(\left\vert \left\vert \sum _{j_{k}}{\varphi }_{(f,j)}(t)\ast {u}_{i}(t)\right\vert -\frac{1}{{t}_{k}{f}_{k}}\sum _{t_{k}}\sum _{f_{k}}\left\vert \sum _{j_{k}}{\varphi }_{(f,j)}(t)\ast {u}_{i}(t)\right\vert \right\vert \right)\right]-x\right\vert \right). \end{eqnarray*}

### Signal reconstruction

To extract the *xHz* part of the powerline noise model, its wavelet transformation is multiplied by the *xHz* portion of the real component of the Morlet wavelet:

(25) \begin{eqnarray*} \displaystyle {d}_{c o m p}(t)=\sum _{j}{\varphi }_{(\mathbf{x}\hspace{0.167em} \boldsymbol{=\hphantom{,} 60}\text{ or }\mathbf{x}\hspace{0.167em} \boldsymbol{=\hphantom{,} 50},j)}(t)\left(\sum _{j_{k}}{\varphi }_{(\mathbf{x}\hspace{0.167em} \boldsymbol{=\hphantom{,} 60}\text{ or }\mathbf{x}\hspace{0.167em} \boldsymbol{=\hphantom{,} 50},j)}(t)\ast {d}_{c o m p}(t)\right).&&\displaystyle \end{eqnarray*}

However, due to the loss of amplitude information after ICA this inversion does not result in an optimal model for denoising. In order to recover the amplitude of the powerline noise, the amplitude of the convolution between the mentioned wavelet at 50/60 Hz center frequency and the denoising component *d_comp_*(*t*) is projected unto that of the original signal *y*(*t*). This projection is accomplished via least squares. An initial powerline noise amplitude prediction is done by normalizing the root-mean-square of the wavelet transformation of the extracted component at *xHz* to that of the wavelet transformation of the original signal, *y*(*t*), at *xHz*.

(26) \begin{eqnarray*} \displaystyle {d}^{{\prime}}(t)&=&\displaystyle {C}^{-1}\frac{\lim _{T\rightarrow \infty }\sqrt{{T}^{-1}\int \nolimits _{0}^{T}{\left[\sum _{j_{k}}{\varphi }_{(x\in S,j)}(t)\ast y(t)\right]}^{2}d t}}{\lim _{T\rightarrow \infty }\sqrt{{T}^{-1}\int \nolimits _{0}^{T}{\left[\sum _{j_{k}}{\varphi }_{(x\in S,j)}(t)\ast {d}_{c o m p}(t)\right]}^{2}d t}}\nonumber\\ \displaystyle &&\displaystyle \times \, \left[\sum _{j}{\varphi }_{(x\in S,j)}(t)\left(\sum _{j_{k}}{\varphi }_{(x\in S,j)}(t)\ast {d}_{c o m p}(t)\right)\right],\nonumber\\ \displaystyle &&\displaystyle \quad \text{ where }S=\{ 50,60\} \wedge C={\left(\sum _{t}\left\vert {\varphi }_{(x\in S,j)}(t)\right\vert \right)}^{2}. \end{eqnarray*}

The powerline noise with the appropriate amplitude is obtained by a regression approach which learns and estimates the parameter *λ* which minimizes the sum of the square distances between the signal and putative powerline noise:

(27) \begin{eqnarray*} \displaystyle A=\mathop{\mathop { \mathrm{argmin}}}\limits _{\lambda \in \{ 0.01,\ldots ,2\} }\sum _{t}{\left(\lambda {d}^{{\prime}}(t)-y(t)\right)}^{2}.&&\displaystyle \end{eqnarray*}

Ultimately, the powerline noise is modeled and the signal is reconstructed as follows:

(28) \begin{eqnarray*} \displaystyle d(t)&=&\displaystyle A{d}^{{\prime}}(t)\nonumber\\ \displaystyle &=&\displaystyle A{C}^{-1}\frac{\lim _{T\rightarrow \infty }\sqrt{{T}^{-1}\int \nolimits _{0}^{T}{\left[\sum _{j_{k}}{\varphi }_{(x\in S,j)}(t)\ast y(t)\right]}^{2}d t}}{\lim _{T\rightarrow \infty }\sqrt{{T}^{-1}\int \nolimits _{0}^{T}{\left[\sum _{j_{k}}{\varphi }_{(x\in S,j)}(t)\ast {d}_{c o m p}(t)\right]}^{2}d t}}\nonumber\\ \displaystyle &&\displaystyle \times \, \left[\sum _{j}{\varphi }_{(x\in S,j)}(t)\left(\sum _{j_{k}}{\varphi }_{(x\in S,j)}(t)\ast {d}_{c o m p}(t)\right)\right],\nonumber\\ \displaystyle \widehat{y(t)}&=&\displaystyle \sum _{i=1}^{k}{c}_{i}(t)+r(t)-d(t)=y(t)-d(t). \end{eqnarray*}

## Additional Information and Declarations

### Data Deposition

The following information was supplied regarding the deposition of related data: https://github.com/akweisek/powerline-noise-elimination.

## References

[ref-1] Agrawal S, Gupta A (2013). Fractal and emd based removal of baseline wander and powerline interference from ecg signals. Computers in Biology and Medicine.

[ref-2] Blanco-Velasco M, Weng B, Barner KE (2008). Ecg signal denoising and baseline wander correction based on the empirical mode decomposition. Computers in Biology and Medicine.

[ref-3] Blaschke T, Wiskott L (2004). Cubica: independent component analysis by simultaneous third-and fourth-order cumulant diagonalization. IEEE Transactions on Signal Processing.

[ref-4] Bloomfield P (2004). Fourier analysis of time series: an introduction.

[ref-5] Brown P (2006). Bad oscillations in Parkinson’s Disease. Journal of Neural Transmission.

[ref-6] Castellanos NP, Makarov VA (2006). Recovering eeg brain signals: artifact suppression with wavelet enhanced independent component analysis. Journal of Neuroscience Methods.

[ref-7] Chang K-M (2010). Arrhythmia ecg noise reduction by ensemble empirical mode decomposition. Sensors.

[ref-8] Chang K-M, Liu S-H (2011). Gaussian noise filtering from ecg by wiener filter and ensemble empirical mode decomposition. Journal of Signal Processing Systems.

[ref-9] Chawla M (2011). Pca and ica processing methods for removal of artifacts and noise in electrocardiograms: a survey and comparison. Applied Soft Computing.

[ref-10] Citi L, Poli R, Cinel C (2010). Modelling and exploiting P300 amplitude changes due to variable target delays in Donchin’s speller. Journal of Neural Engineering.

[ref-11] Clancy E, Morin EL, Merletti R (2002). Sampling, noise-reduction and amplitude estimation issues in surface electromyography. Journal of Electromyography and Kinesiology.

[ref-12] Cui H-F, Ye J-S, Chen Y, Chong S-C, Sheu F-S (2006). Microelectrode array biochip: tool for in vitro drug screening based on the detection of a drug effect on dopamine release from pc12 cells. Analytical Chemistry.

[ref-13] Daubechies I (1990). The wavelet transform, time-frequency localization and signal analysis. IEEE Transactions on Information Theory.

[ref-14] De-xiang Z, Xiao-pei W, Xiao-jing G (2008). The eeg signal preprocessing based on empirical mode decomposition. The 2nd international conference on bioinformatics and biomedical engineering, 2008. ICBBE 2008.

[ref-15] Ferdjallah M, Barr RE (1990). Frequency-domain digital filtering techniques for the removal of powerline noise with application to the electrocardiogram. Computers and Biomedical Research.

[ref-16] Ferdjallah M, Barr RE (1994). Adaptive digital notch filter design on the unit circle for the removal of powerline noise from biomedical signals. IEEE Transactions on Biomedical Engineering.

[ref-17] Finnis KW, Starreveld YP, Parrent AG, Sadikot AF, Peters TM (2003). Three-dimensional database of subcortical electrophysiology for image-guided stereotactic functional neurosurgery. IEEE Transactions on Medical Imaging.

[ref-18] Garcia-Gonzalez MA, Argelagos-Palau A, Fernandez-Chimeno M, Ramos-Castro J (2013). A comparison of heartbeat detectors for the seismocardiogram.

[ref-19] Gerson MC, Phillips JF, Morris SN, McHenry PL (1979). Exercise-induced u-wave inversion as a marker of stenosis of the left anterior descending coronary artery. Circulation.

[ref-20] Goldberger A, Amaral L, Glass L, Hausdorff J, Ivanov P, Mark R, Mietus J, Moody G, Peng C-K, Stanley H (2000). PhysioBank, PhysioToolkit, and PhysioNet: components of a new research resource for complex physiologic signals. Circulation.

[ref-21] Guridi J, Gorospe A, Ramos E, Linazasoro G, Rodriguez MC, Obeso JA (1999). Stereotactic targeting of the globus pallidus internus in parkinsons disease: imaging versus electrophysiological mapping. Neurosurgery.

[ref-22] Hamilton PS (1996). A comparison of adaptive and nonadaptive filters for reduction of power line interference in the ecg. IEEE Transactions on Biomedical Engineering.

[ref-23] Huang NE, Shen Z, Long SR, Wu MC, Shih HH, Zheng Q, Yen N-C, Tung CC, Liu HH (1998). The empirical mode decomposition and the hilbert spectrum for nonlinear and non-stationary time series analysis. Proceedings of the Royal Society of London. Series A: Mathematical, Physical and Engineering Sciences.

[ref-24] Hyvärinen A, Karhunen J, Oja E (2004). Independent component analysis.

[ref-25] Iriarte J, Urrestarazu E, Valencia M, Alegre M, Malanda A, Viteri C, Artieda J (2003). Independent component analysis as a tool to eliminate artifacts in eeg: a quantitative study. Journal of Clinical Neurophysiology.

[ref-26] Jain T, Muthuswamy J (2008). Microelectrode array (mea) platform for targeted neuronal transfection and recording. IEEE Transactions on Biomedical Engineering.

[ref-27] Jones IL, Livi P, Lewandowska MK, Fiscella M, Roscic B, Hierlemann A (2011). The potential of microelectrode arrays and microelectronics for biomedical research and diagnostics. Analytical and Bioanalytical Chemistry.

[ref-28] Keshtkaran MR, Yang Z (2012). Power line interference cancellation in in-vivo neural recording.

[ref-29] Keshtkaran MR, Yang Z (2014). A fast, robust algorithm for power line interference cancellation in neural recording. Journal of Neural Engineering.

[ref-30] Kuzilek J, Kremen V, Lhotska L (2014). Comparison of jade and canonical correlation analysis for ecg de-noising.

[ref-31] Kuzilek J, Kremen V, Soucek F, Lhotska L (2014). Independent component analysis and decision trees for ecg holter recording de-noising. PLoS ONE.

[ref-32] Levkov C, Mihov G, Ivanov R, Daskalov I, Christov I, Dotsinsky I (2005). Removal of power-line interference from the ecg: a review of the subtraction procedure. BioMedical Engineering OnLine.

[ref-33] Li G, Zeng X, Zhou X, Zhou Y, Liu G, Zhou X (2012). Robust suppression of nonstationary power-line interference in electrocardiogram signals. Physiological Measurement.

[ref-34] Mariyappa N, Sengottuvel S, Patel R, Parasakthi C, Gireesan K, Janawadkar M, Radhakrishnan T, Sundar C (2015). Denoising of multichannel mcg data by the combination of eemd and ica and its effect on the pseudo current density maps. Biomedical Signal Processing and Control.

[ref-35] Miwa K, Miyagi Y, Fujita M, Fujiki A, Sasayama S (1993). Transient terminal u wave inversion as a more specific marker for myocardial ischemia. American Heart Journal.

[ref-36] Mizuseki K, Sirota A, Pastalkova E, Buzsáki G (2009). Multi-unit recorings from the rat hippocampus made during open field foraging.

[ref-37] Naji M, Firoozabadi M, Kahrizi S (2011). The application of empirical mode decomposition in elimination of ecg contamination from emg signals.

[ref-38] Nimunkar AJ, Tompkins WJ (2007). Emd-based 60-hz noise filtering of the ecg.

[ref-39] Oweiss KG (2010). Statistical signal processing for neuroscience and neurotechnology.

[ref-40] Philips W (1996). Adaptive noise removal from biomedical signals using warped polynomials. IEEE Transactions on Biomedical Engineering.

[ref-41] Piskorowski J (2012). Powerline interference removal from ecg signal using notch filter with non-zero initial conditions.

[ref-42] Poornachandra S, Kumaravel N (2008). A novel method for the elimination of power line frequency in ecg signal using hyper shrinkage function. Digital Signal Processing.

[ref-43] Poungponsri S, Yu X-H (2013). An adaptive filtering approach for electrocardiogram (ecg) signal noise reduction using neural networks. Neurocomputing.

[ref-44] Schalk G, McFarland DJ, Hinterberger T, Birbaumer N, Wolpaw JR (2008). A general-purpose brain-computer interface (BCI) system. IEEE Transactions on Biomedical Engineering.

[ref-45] Schramm J, Koht A, Schmidt G, Pechstein U, Taniguchi M, Fahlbusch R (1990). Surgical and electrophysiological observations during clipping of 134 aneurysms with evoked potential monitoring. Neurosurgery.

[ref-46] Stett A, Egert U, Guenther E, Hofmann F, Meyer T, Nisch W, Haemmerle H (2003). Biological application of microelectrode arrays in drug discovery and basic research. Analytical and Bioanalytical Chemistry.

[ref-47] Van Alste J, Schilder T (1985). Removal of base-line wander and power-line interference from the ecg by an efficient fir filter with a reduced number of taps. IEEE Transactions on Biomedical Engineering.

[ref-48] Van Eck HJR, Kors JA, Van Herpen G (2005). The u wave in the electrocardiogram: a solution for a 100-year-old riddle. Cardiovascular Research.

[ref-49] Wang Y-H, Yeh C-H, Young H-WV, Hu K, Lo M-T (2014). On the computational complexity of the empirical mode decomposition algorithm. Physica A: Statistical Mechanics and its Applications.

[ref-50] Wu Z, Huang NE (2009). Ensemble empirical mode decomposition: a noise-assisted data analysis method. Advances in Adaptive Data Analysis.

[ref-51] Xue Z, Li J, Li S, Wan B (2006). Using ica to remove eye blink and power line artifacts in eeg.

[ref-52] Zhang X, Zhou P (2013). Filtering of surface emg using ensemble empirical mode decomposition. Medical Engineering & Physics.

[ref-53] Zivanovic M, González-Izal M (2013). Simultaneous powerline interference and baseline wander removal from ecg and emg signals by sinusoidal modeling. Medical Engineering & Physics.

